# Limiting heterogeneity in cross-border data flow: Impact on domestic value chains stability and the role of innovation

**DOI:** 10.1371/journal.pone.0308716

**Published:** 2024-08-19

**Authors:** Yang Liu, Yuan Zhang, Rui Jiang, Jing Cheng, JingJing Dai

**Affiliations:** 1 School of Economics and Management, Kunming University, Kunming, Yunnan, China; 2 School of Architecture and Engineering, Yunnan Agricultural University, Kunming, Yunnan, China; University of Agriculture Faisalabad, PAKISTAN

## Abstract

Amidst growing skepticism towards globalization and rising digital trade, this study investigates the impact of Restrictions on Cross-Border Data Flows (RCDF) on Domestic Value Chains (DVCs) stability. As global value chains participation declines, the stability of DVCs—integral to internal economic dynamics—becomes crucial. This study situates within a framework exploring the role of innovation and RCDF in the increasingly interconnected global trade. Using a panel data fixed effect model, our analysis provides insights into the varying effects of RCDF on DVCs stability across countries with diverse economic structures and technological advancement levels. This approach allows for a nuanced understanding of the interplay between digital trade policies, value chain stability, and innovation. RCDF tend to disrupt DVCs by negatively impacting innovation, which necessitates proactive policy measures to mitigate these effects. In contrast, low-income countries experience a less detrimental impact; RCDF may even aid in integrating their DVCs into Global Value Chains, enhancing economic stability. It underscores the need for dynamic, adaptable policies and global collaboration to harmonize digital trade standards, thus offering guidance for policy-making in the context of an interconnected global economy.

## Introduction

In the contemporary global landscape, profound transformations are reshaping the fabric of international trade and economics. A notable trend in this era is the burgeoning skepticism towards globalization, compelling nations to reassess the intricate interplay between their international trade strategies and domestic economic imperatives. This evolving stance has precipitated increased trade tensions and barriers, leading to significant disruptions in global value chains (GVCs) [1]. This is evidenced by a steady decline in GVCs participation since 2012, according to World Bank data [2], reflecting challenges to the efficacy and advancement of value chain processes.

Central to understanding these global shifts is the interplay between GVCs and domestic value chains (DVCs) [3]. DVCs, encompassing the distribution of labor and the exchange of goods within a country [4, 5], are pivotal for a country’s internal economic dynamics. The stability of DVCs referring to their capacity to adapt and maintain efficiency despite external disruptions, is now more important than ever in fostering higher stability in global trade, particularly considering the diverse impacts on countries at different stages of development. The impact of globalization, digital trade, and the RCDF on GVCs and DVCs can vary considerably among countries with varying levels of economic structure and technological advancement. For example, developing countries may face unique challenges in integrating into GVCs [6], while developed countries may be more focused on technological innovation and digitalization. Understanding these nuances is crucial for the formulation of targeted policies that address the specific needs and constraints of countries at different stages of development.

This study situates these developments within a broader theoretical framework, tracing the evolution of the value chain concept from its inception [7] to the contemporary notions of the “global commodity chain” [8] and “global value chain”[9]. Our focus extends from key factors affecting the position of GVCs, such as factor endowment [10–12], financial development [13], and institutional stability [14], to innovation and RCDF, which underline the increasing complexity and interconnectedness in global trade dynamics, especially under the influence of digitalization.

We explore the context of the burgeoning trade in digital trade and trade in digital services, collectively referred to as the exchange of goods and services through digital means [15], as well as those provided via digital platforms and technologies [16]. This exploration is crucial as it sheds light on how data factor flows—a key component of digital trade—affect the dynamics of the value chain. The boom of digital services in international trade is not only a trend, but also a transformative force [17, 18], making RCDF a critical factor in affecting the stability of DVCs [15].

In response to these global economic shifts, numerous nations are recalibrating their trade strategies, with a particular emphasis on bolstering their DVCs to mitigate risks associated with participation in GVCs. The intricate interplay between RCDF and the stability of DVCs significantly impacts the progression of digital trade, subsequently influencing the dynamics of GVCs [19]. This strategic recalibration necessitates a critical examination of cross-border data flow restrictions. Central to this inquiry are pivotal questions: Does limiting these data flows enhance the stability of DVCs, or does it, in contrast, hinder their integration with GVCs? Furthermore, assessing whether the effects of data flow restrictions on the stability of DVCs are consistent across economies at different developmental stages is crucial. Considering the significant repercussions of these issues on both regional collaboration and the broader spectrum of global trade dynamics, an in-depth and nuanced exploration is indeed imperative.

This paper sets out to comprehensively address the complexities and dynamics of digital trade policies, aiming to contribute significantly both in theoretical frameworks and practical applications.

- Theoretically, central to this study is the thesis that prioritizing “stability” over “efficiency” in value chain research is essential. The study endeavors to elucidate the principles underpinning shifts in digital services trade policies and their effects on DVCs stability, particularly highlighting the role of innovation. It introduces a novel perspective by examining how RCDF influences the robustness and resilience of DVCs, employing a methodology that utilizes each country’s input-output table—a technique not extensively explored in previous literature.
- Practically, the study offers insightful policy recommendations for countries to effectively leverage industrial digitalization and maximize digital trade benefits. It aims to lay a foundational basis for broader international economic and trade cooperation, potentially contributing to a “re-globalization” movement.
- Empirically, employing a panel data fixed effect model, the study probes the effects of RCDF on the operational dynamics of DVCs. This includes analyzing mechanisms from an innovation perspective and providing a reference for establishing diverse and stable international economic and trade relations. The study also examines the differentiated consequences of RCDF on countries at various income levels, aiming to provide insights that could support and enhance international economic and trade cooperation.

Ultimately, the research aims to empower nations with essential insights for effectively navigating the digital era. It fosters global collaboration across various sectors, contributing to the development of stable international economic and trade relations, especially in the context of advancing “anti-globalization”.

## Literature review

The ongoing digital transformation of the global economy, a key feature of the Fourth Industrial Revolution as described by Schwab (2017) [20], has significant implications for international trade and global commerce [21–23]. Vial (2019) indicates the importance of digital transformation as a process in which the use of digital technologies causes disruptions, leading organizations to respond strategically by changing their methods of creating value [23]. ElMassah and Mohieldin (2020) highlight this transformation through the lens of localizing sustainable development goals, emphasizing the digitization of production and business processes as a pivotal aspect [21]. This shift is leading to a reconfiguration of international trade, characterized by the migration of many operations to the digital realm and a trend towards localized trade due to distributed manufacturing and decentralized value chains [1]. Jouanjean et al. (2019) delves into the effects of digitalization on the agriculture and food sectors, demonstrating how it influences the distribution among value chain participants and reduces trade and transaction costs [24]. While these developments mark significant progress, they are not without challenges. Aaronson (2023) introduces a critical perspective, pointing out the regulatory challenges presented by this digital revolution. Particularly, governments are imposing restrictions on global data flows, undermining the economic benefits of digital trade [25]. This juxtaposition of advancement and regulation underscores the complex nature of the digital transformation in the global economy.

Focusing on digital trade barriers, recent studies emphasize their unique challenges to global commerce [26–28]. Meltzer (2019) explores the governance aspects of digital trade, emphasizing the complex nature of these emerging barriers [29]. Potluri et al. (2020) discuss the effects of data localization on digital trade, arguing that such barriers often protect established firms at the expense of consumers [28]. Jiang et al. (2022) further provide empirical evidence on how digital trade barriers, specifically in the context of China, impact export performance by constraining service inputs and hindering foreign direct investment (FDI) entry [26]. In the broader context of GVCs, the impact of trade barriers is a critical area of investigation [16, 30–32]. Hufbauer et al. (2013) study the impact of trade barriers on the participation of exporting countries, highlighting that these barriers hinder the smooth functioning of the value chain [30]. Biryukova and Vorobjeva (2017) examine the impact of service liberalization on the participation of BRICS countries in GVCs, revealing that service trade restrictions significantly hinder this process [32]. Contrary to the expectation that trade restrictions lead to GVCs’ shrinkage or disruption, Gereffi et al. (2021) posit that the unintended consequences of trade policies, both restrictive and liberal, are exacerbated by the complexity and prevalence of GVCs [31]. Wang et al.(2023) further demonstrate empirically that digital trade rules significantly influence forward GVCs [16].

Further extending the discussion to RCDF, their far-reaching effects on GVCs are explored in recent studies. Studies such as those by Ferracane et al. (2018) have explored the extensive ramifications of RCDF on GVCs [33]. These studies predominantly focus on how such restrictions influence international trade patterns, alter global production and service distribution within GVCs, and affect market entry strategies and innovation capacities across different economies. Lastly, Gereffi et al. (2021) discuss the effects of anti-globalization policies, including various barriers, on value-added activities in GVCs. They argue that these policies can disrupt GVCs by restricting the flow of knowledge, thereby affecting the overall dynamics of GVCs [31].

The preceding discussion reveals two primary research gaps. Firstly, there is a noticeable absence of studies that specifically address the impact of RCDF on the stability of DVCs across various countries. Given the pivotal role of DVCs in an increasingly anti-globalizations economy, particularly for low-income countries, it is imperative to explore whether the effects of RCDF mirror those of traditional barriers and if these impacts are consistent across different economic contexts. Furthermore, it is equally important, yet less explored, how RCDF affect DVCs, considering their intricate interconnections with GVCs. This aspect is crucial for understanding the full spectrum of the digital trade barriers’ impact. Secondly, while the current body of research has provided valuable insights into the effects of digital trade barriers at a bilateral or regional level, there is a significant gap in understanding these dynamics from a more global perspective, encompassing a diverse range of countries and industries. Unraveling the nuances of how RCDF influences DVCs on a global scale, especially in terms of value-added production, is crucial for comprehensively understanding the broader implications of digital trade barriers.

## Theoretical analysis

RCDF, conceptualized as trade costs, directly impede the free movement of digital factors, which are integral to the efficiency of GVCs [34, 35]. These barriers engender a rise in operational costs for businesses, exemplified by increased search costs for trade intermediaries, heightened transaction costs in production’s division, and the added burden of navigating bidirectional regulatory compliance​ [36, 37], The ripple effect of these increased costs may prompt industries to consider domestic relocation, thereby disrupting the established synergies between domestic and foreign digital factors within GVCs, which in turn jeopardizes the stability of DVCs.

The ramifications of RCDF are multifaceted. They not only inflate operational costs and disturb existing interdependencies but also provoke potential industry relocations within GVCs [38]. This disturbance diminishes market share and the capacity for rapid adaptation in dynamic markets, further constraining decision-making agility and the dynamics of relationships—both of which are indispensable for maintaining the viability and strategic flexibility of service systems [39].

Moreover, the role of digital technologies as specialized factors in production division is integral to both production efficiency and management acumen [40, 41], When RCDF interrupt the digital empowerment of DVCs, the synergy and collaboration between upstream and downstream enterprises deteriorate. This breakdown leads to a compromised supply of intermediate goods and a consequent dip in DVCs stability [33, 42, 43]. Such an inhibition of technological innovation sends shockwaves throughout the system [44], indirectly crippling a country’s capacity for innovation and, as a result, the enhancement of processes within DVCs. The domino effect continues as this leads to a diminished production and service capacity within enterprises [33, 45], eroding the stability of intermediate goods supply. Furthermore, RCDF thwart the progression of DVCs into sectors that yield higher value-added products [46, 47], thereby diluting inter-industry production linkages [48] and further destabilizing DVCs.

Based on this nuanced analysis, the paper firmly posits the following hypothesis:

Hypothesis 1: RCDF inhibit the stability of DVCs.
Hypothesis 2: The impact of RCDF on DVCs stability is mediated by their stifling effect on technological innovation, which is essential for the continuous improvement and value addition within these chains.

According to the Hicks Theorem, which posits that technology improvements in industries where a country lacks a comparative advantage lead to welfare reductions in foreign nations [49], advancements in digital technology have, however, narrowed global labor cost disparities [50]. A critical factor influencing firms’ participation in DVCs in international markets is the economic disparity between home and host countries. Nations at varying economic levels exhibit significant differences in resource quality and cost [51], impacting their roles within DVCs. impacting their roles within DVCs. Developed economies, such as the United States, Japan, and Germany, with their advanced digital technologies, provide digital intermediates and services globally [52]. This positions them at the higher-value segments of DVCs, such as design, innovation, and analytics. In contrast, low-income countries, constrained by their lesser digital capabilities and infrastructure, often participate in lower-value segments, such as assembly or basic data processing.

On one hand, changes in factor endowments, spurred by digital service trade, can transform value chain logic [53], allowing affluent nations to enhance their trade terms and facilitate DVCs’ upgrading. Paradoxically, RCDF enforced by these nations may hinder the enhancement of their own DVCs stability. Conversely, these countries could use their digital dominance to expand the digital divide, erecting developmental barriers for other nations [54]. Meanwhile, low-income countries, grappling with the impacts on both traditional and digital sectors, may find increasing RCDF instrumental in protecting their comparative advantages and stabilizing their DVCs. This observation leads to Hypothesis 3:

Hypothesis 3: The impact of RCDF on DVCs varies among countries (regions) with different income levels.

**Fig 1:**
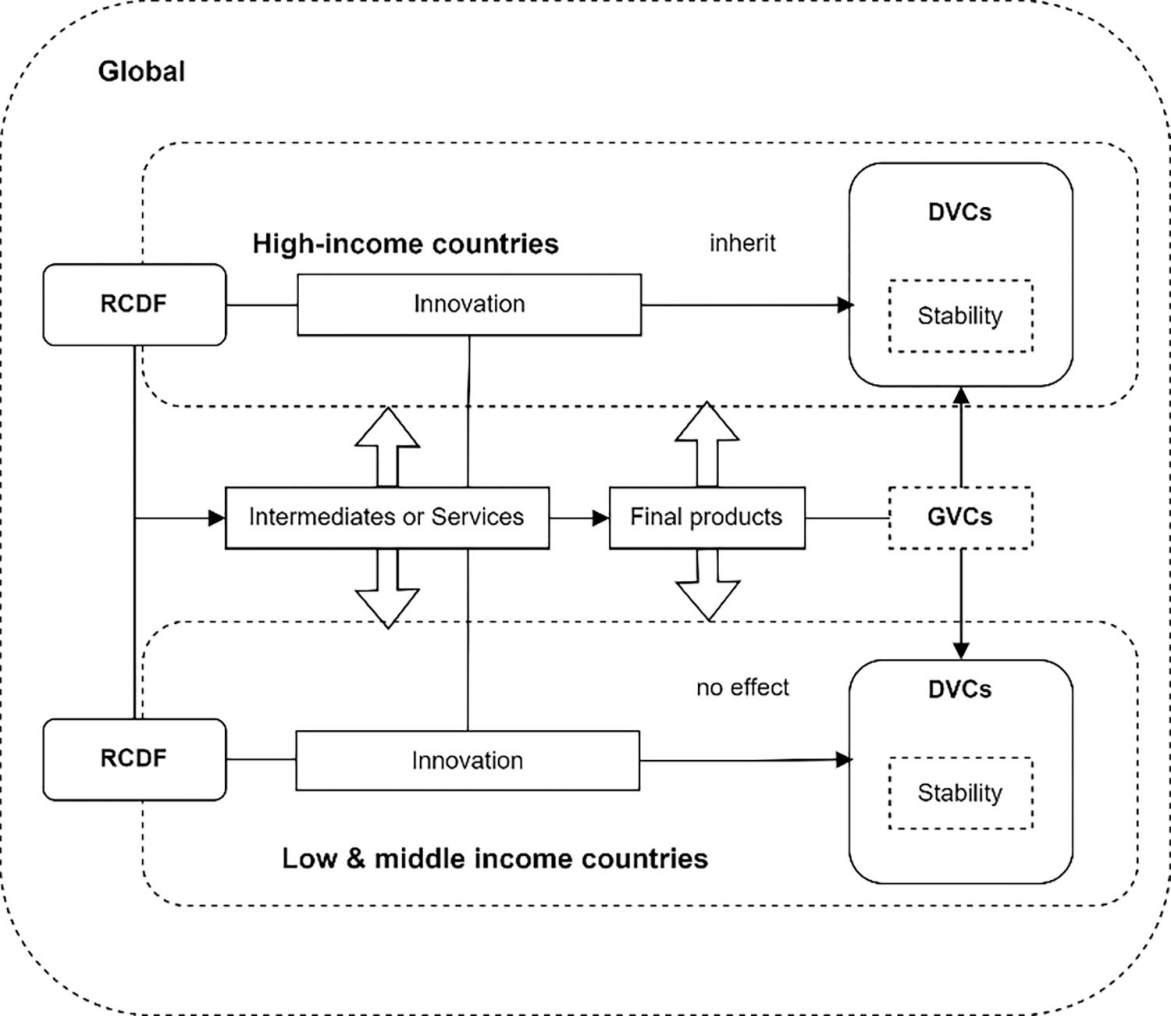
Theoretical analysis framework.

In summary, RCDF obstruct transaction efficiency, impede the formation and integration of DVCs, and hinder the transition from traditional to innovative industry dynamics within GVCs. These limitations directly erode the confidence and willingness of traders to export, disrupt the acquisition of intermediate goods in deeply involved industries, and challenge DVCs stability. Additionally, by curtailing technological innovation within value chains and escalating trade costs, RCDF adversely affect intermediate goods supply levels, further compromising DVCs stability. Consequently, the paper’s theoretical analysis framework, refer to Fig 1, emerges from these findings.

## Material and methods

### Model

This study constructs a three-dimensional panel data set that includes “country (region)-industry-year” and establishes the following econometric model to test the first hypothesis:

$$DVCs_{\theta it}=\beta_{0}+\beta_{1}RCDF_{\theta it}+\beta_{2}\mathrm{Controls}+v_{\theta }+v_{i}+v_{t}+\varepsilon_{\theta it}$$ (1)

In Eq (1), θ,*i*,*t* represent country (region), industry, and year, respectively. The dependent variable *DVCs*_*θit*_ signifies the DVCs stability in the given country for that year. The core independent variable *RCDF*_*θit*_ represents the level of RCDF in the industry in the given country for that year. Controls denote the control variables. *v*_*θ*_, *v*_*i*_, and *v*_*t*_ represent fixed effects for country (region), industry, and year, respectively. *ε*_*θit*_ is a random error term.

### Variable settings

#### DVCs stability

In this study, we follow the methodology outlined by Wang et al. (2017) [55], which involves using the decomposition matrix to analyze the value added by each industry within a country. This approach enables us to calculate the domestic value added consumed in the production of final products within a country. It is important to recognize that the flow of the value chain is intricately linked with the incorporation of intermediate goods throughout DVCs. Hence, the stability of the production and supply of these intermediate goods within the value chain is critical to the overall stability of DVCs.

To quantitatively evaluate the stability of DVCs, our study adopts the metric proposed by Baldwin and Gu (2009) [56], which is the inverse of production volatility. This approach essentially inverses the fluctuations in production levels, providing a more stable measure. A lower volatility score, hence a higher inverse value, indicates greater consistency and reliability in the production processes within DVCs. This makes it a valuable indicator for assessing the overall stability of DVCs. By employing this metric, we gain deeper insights into how smoothly and predictably the production elements of DVCs operate, which is crucial for understanding the robustness and resilience of DVCs in a dynamic global economy.

$${DVCs}_{\theta j}^{n}=\sum_{i\in n}\left(\frac{1}{sdBP_{\theta i}^{n}}\times \frac{DVA_{\theta \dot{j}}^{nn}}{Y_{\theta j}^{n}}\right)$$ (2)

where ${DVCs}_{\theta j}^{n}$ denotes the stability of DVCs in the industry of country j in θ. $\frac{DVA_{\theta \dot{j}}^{nn}}{Y_{\theta j}^{n}}$ denotes inter-industry production linkages, measured as the ratio of value added consumed by industry production $DVA_{\theta \dot{j}}^{nn}$ to the value of the final product $Y_{\theta j}^{n}$. The term $\frac{1}{sdBP_{\theta i}^{n}}$ denotes the stability of the supply of intermediate goods in industry j of country θ. This paper measures it using the reciprocal of the fluctuation rate of the average production length. The average production length refers to the average number of times the added value created by production factors in the sequential production process is counted as total output [55]. Essentially, it reflects the degree of closeness between industries. The smaller the fluctuation of the average production length, the more stable the closeness between industries, and the higher the stability of the supply of intermediate goods. The calculation formula is as follows:

$$BP_{\theta jt}=B_{\theta jt}-\overset{——}{B_{\theta t}}$$ (3)

$$sdBP_{\theta jt}=\sqrt{\frac{1}{T-1}\sum_{i=1}^{T}{\left(BP_{\theta ij\tau }-\frac{1}{T}\sum_{t=1}^{T}BP_{\theta ij\tau }\right)}^{2}}$$ (4)

where *BP*_θ*ji*_ denotes the backward average production length of industry j in country θ, and *B*_θ*jt*_ denotes the backward average production length of industry j in year t; $\overset{——}{B_{\theta t}}$ is the backward average production length of all industries. *sdBP*_θ*jt*_ denotes the backward averaged length of production in the *τ* observation period excluding systematic risk; set 3 years as an observation period, and the last year 2020 is calculated as a moving average.

#### RCDF

This study employs data from the OECD’s Digital Services Trade Restrictiveness Index (DSTRI) [57] to represent the level of RCDF. The DSTRI methodology adopts a binary system for its analysis, where a score of 1 indicates the presence of industry-specific restrictions, and a score of 0 signifies their absence. The weighting of the index is based on evaluations made by experts in the OECD database. These experts assess the relative significance of five domains: publicly available laws and regulations, policy information, and restrictive measures. The indicators, as derived from this method, vary between 0 and 1. A higher score on this scale denotes more significant restrictions on digital service trade for a given country or region.

#### Controlling variables

In evaluating the impact of RCDF on stability of DVCs, it is essential to incorporate control variables for a more accurate and effective analysis. These variables, drawn from previous studies, include:

- Industry Foreign Direct Investment Regulatory Level (FDIR): The regulatory environment for foreign direct investment (FDI) significantly affects multinational corporations, which act as bridges between domestic and global value chains. Stringent FDI regulations can increase the costs and risks associated with investment and trade, and reduce transaction efficiency. Consequently, tougher FDI rules may discourage these corporations from investing and trading, adversely affecting stability of DVCs.
- Economic Development (ED): The economic strength of a country influences its market depth, with deeper markets facilitating more intra-industry trade and a more sophisticated value chain system. Countries with higher levels of economic development are more likely to engage in “task trade”, enhancing stability of DVCs. This study employs the economic development index from the SPI database as a measure of ED. This selection is made because the index offers a comprehensive account of the economy’s development, encompassing a broad spectrum of economic indicators and factors.
- Digital Technology Development (DTD): Countries with advanced digital technology are often able to source labor-intensive products or services from global value chains, shifting towards more technology- or capital-intensive production processes domestically. This transition improves the stability of DVCs. In this study, the data product index from the SPI database serves as a proxy for digital technology development, where a higher index signifies superior technological research and effective application of digital technology.
- Trade Openness (TO): A country’s trade openness reflects its integration into the global value chain. Economies that are more open tend to have stronger domestic production capabilities and higher stability of DVCs. In this study, the TO and FO index from the KOF database serves as a proxy for trade openness and financial openness.
- Financial Openness (FO): A country’s openness in trade and finance can help to break down the monopoly patterns of interest groups, thereby enhancing the quality of domestic value chains. Therefore, this indicator is also included as a control variable in our study.

### Data sources

This study utilizes a comprehensive dataset spanning from 2014 to 2020, drawing from multiple databases and sources. The WIOD-SEA database provided the sub-industry employment data for 2014, while data from 2015 onwards was segmented and processed based on the input-output share. The total labor force data was sourced from the WDI database.

Various other databases were instrumental in gathering specific information. The UIBE GVCs database and the ADB input-output database were used to derive data on industry value added, final product value added, industry production consumption added, and backward average length of production. The OECD database supplied the digital cross-border flow restriction index and the industry FDI regulation index. The SPI database contributed the employment economic development index and the digital product index. To measure the level of trade openness and financial openness, data was extracted from the KOF database and the World Bank. These databases provided detailed information on these specific variables. For further details on these variables, refer to Table 1.

**Table 1:** Variable description.

Variable Abbreviation	Measurement Indicator	Data Description	Source
*lnDVCs*	DVCs stability	Backward Average Production Length	World Bank WDI (2022)
*lnRCDF*	RCDF	DSTRI [57]: The DSTRI methodology uses a binary system, scoring “Yes” or “No” for trade measures (0 for no restrictions, 1 for restrictions), and accounts for specific regulatory and market features, as well as measure interrelations.	OECD Database [57]
*lnFDIR*	Industry FDI regulation level	FDI Regulatory Restrictiveness Index [58]: The OECD Foreign Direct Investment Regulatory Restrictiveness Index (FDI RRI) is a composite measure, ranging from 0 to 1, that quantifies statutory restrictions on FDI across four dimensions: foreign equity limitations, screening/approval requirements, rules for key personnel, and operational restrictions for foreign enterprises.	OECD Database [59]
*lnED*	Economic Development Level	Employment Economic Development Index	SPI Database [60]
*lnDTD*	Digital Technology Development Level	Digital Product Index	
*lnTO*	Level of trade openness	Share of trade in goods (%GDP) weights: 38.1%	World Bank WDI (2022)
		Share of trade in services (%GDP) weights: 42.6%	World Bank WDI (2022)
		Herfindahl-Hirschman Index weights: 19.3%	IMF DOTS (2022)
*lnFO*	Level of financial openness	Sum of stocks of assets and liabilities of foreign direct investments (% of GDP) weights: 26.3%	Lane and Milesi-Ferretti (2018) [61] and IMF IIP (2022)
		Sum of stocks of assets and liabilities of international equity portfolio investments (% of GDP) weights: 16.5%	
		Sum of inward and outward stocks of international portfolio debt securities and international bank loans and deposits (% of GDP) weights: 29%	
		Includes foreign exchange (excluding gold), SDR holdings and reserve position in the IMF (% of GDP) weights: 0.8%	
		Sum of capital and labour income to foreign nationals and from abroad (% of GDP) weights: 27.5%	World Bank WDI (2022)

### Descriptive statistics

Table 2 shows the descriptive statistics of all variables including sample size, mean, variance and maximum and minimum values.

**Table 2:** Descriptive statistics.

Variable name	Observed value	Mean	Standard deviation	Minimum	Maximum
*lnDVCs*	11137	3.464	2.770	-32.919	39.777
*lnRCDF*	11515	-2.132	1.681	-16.118	-0.435
*lnFDIR*	11515	-11.674	6.429	-16.118	0.000
*lnED*	11515	-0.458	0.298	-1.790	0.021
*lnDTD*	11515	4.182	0.202	3.301	4.562
*lnTO*	11515	4.318	0.269	3.584	4.543
*lnFO*	11515	4.276	0.302	3.178	4.605

As depicted in Table 2, it can be seen that the mean value of the explanatory variables is 3.464, the maximum value is 39.777, the minimum value is -32.919, and the standard deviation is 2.770, which is not much different from the mean value, indicating that there is no over-dispersion after taking the logarithm. The core explanatory variable has a standard deviation of 1.681 and a mean of -2.132, indicating that the distribution of RCDF vary greatly across countries. This may lead to a repositioning of firms’ production activities to avoid the efficiency impacts of production and trade caused by different barriers [62], which in turn leads to the segmentation of the value chain.

## Results

### Benchmark regression analysis

In Table 3, the baseline regression results (M1) and the post-shrinkage results (M2) illustrate the effect of RCDF on the stability of DVCs. In M1, the negative coefficient of RCDF at -0.0385, significant at the 1% level, indicates a detrimental impact on DVCs stability. This finding is aligned with Hypothesis 1, suggesting that RCDF may act as a barrier at the interface of GVCs and DVCs. It appears to disrupt the stable supply of intermediate products and the cohesion of production linkages within the value chain, ultimately impeding the enhancement of the DVCs

**Table 3:** Regression results.

Viable	M1	M2	high-income group	low-income group
			M3	M4
*lnRCDF*	-0.0385*** (-3.03)	-0.0302* (-2.54)	-0.0358*** (-2.70)	0.678** (2.01)
*lnFDIR*	-0.0163*** (-3.18)	-0.0173*** (-4.33)	-0.0195*** (-3.24)	0.0092 (1.07)
*lnED*	0.624*** (3.83)	0.655*** (4.65)	1.183*** (3.30)	0.288* (1.71)
*lnDTD*	-1.722*** (-3.90)	-1.511*** (-4.36)	-3.056*** (-5.20)	2.926*** (4.24)
*lnTO*	1.978*** (4.08)	1.866*** (4.32)	2.686*** (3.48)	2.179*** (3.02)
*lnFO*	2.339*** (3.15)	1.918*** (2.98)	2.982*** (3.46)	1.950 (1.14)
_cons	-7.862 (-1.60)	-6.441 (-1.48)	-8.364 (-1.32)	-23.32** (-2.48)

Year	Yes	Yes	Yes	Yes
Country	Yes	Yes	Yes	Yes
Industry	Yes	Yes	Yes	Yes
N	11137	11137	9486	1651
R-sq	0.375	0.474	0.384	0.415

Note:

***, **, and * indicate significant at the 1%, 5% and 10% levels, respectively, with T-values in parentheses.

This interpretation of RCDF’s impact is more profound when contrasted with earlier studies. For instance, Meltzer (2015) observed a more moderate impact of RCDF on international trade [47], indicating a potential evolution in the nature and effect of RCDF over time. This evolution could be attributed to the increasing complexity and integration of digital factors in global trade.

Regarding the coefficients of other continuous variables, the negative coefficient of industry FDI regulation in M1 (-0.0163) at a significant level suggests that more relaxed FDI regulations are conducive to creating value chains with higher value-added, likely due to enhanced technological spillovers and capital accumulation from FDI. This observation is consistent with the findings of Martínez-Galán and Fontoura (2015), which also underscored the positive influence of liberal FDI policies on value chain enhancement [63].

Economic development (lnED), showing a positive and significant coefficient (0.624) in M1, reaffirms that higher economic development is a crucial factor in augmenting DVCs stability. Furthermore, the positive coefficients for trade openness (lnTO) (1.978) and financial openness (lnFO) (2.339) underscore the importance of global integration and diverse capital supply in enhancing DVCs production levels, echoing the conclusions of Gereffi (2015) on the benefits of globalization and financial liberalization for value chain division [64].

Interestingly, the negative coefficient of digital technology development (lnDTD) suggests a counterintuitive impact on DVCs stability. This may imply that while digital technologies offer numerous advantages, their rapid development and integration might lead to a restructuring of value chains that temporarily destabilizes existing setups. This finding echoes Kehoe and Mateer (2015), which concluded that digital technology negatively affects the distribution segment of an industry-specific value chain [65, 66], indicating a more nuanced relationship that warrants further investigation.

In the segmented analysis for high-income (M3) and low-income (M4) countries, the contrasting coefficients of RCDF reveal distinct impacts based on economic status. In high-income regions, the negative coefficient of RCDF (-0.0358) at the 1% significant level suggests that RCDF significantly suppresses the stability of DVCs. This could be attributed to the advanced digital infrastructures in these regions, where RCDF may hinder the optimal utilization of digital technologies in value chains, thus reducing labor’s relative contribution and possibly leading to a decrease in labor productivity. This finding supports from the assertions of studies like Meltzer (2019), who observed that RCDF undermined the economic benefits of digital trade [29].

Contrastingly, in low-income regions, the positive coefficient of RCDF (0.678) at the 5% significant level presents a different scenario. The increase in RCDF appears to contribute positively to the stability of DVCs. This can be interpreted as RCDF acting as a protective measure for domestic industries, particularly in economies where digital infrastructure is less developed. By limiting the influx of advanced digital services and products, RCDF allows local industries more time to adapt and integrate into the evolving digital global economy. This finding diverges from the assertions of studies like Dong et al. (2020), which hypothesized a negative impact of RCDF on the competitiveness of digital trade in all economies [67].

This nuanced understanding of RCDF’s impact is crucial, as it highlights the divergent pathways through which digital trade policies affect economies at different stages of development. In high-income countries, where digital services and technologies are deeply embedded in the value chain, restrictions can disrupt established patterns and impede efficiency. However, in low-income countries, where the integration of digital technologies is still in nascent stages, these restrictions can provide a buffer, allowing for a more gradual integration into the global digital economy. The implications of these findings are significant, particularly in the context of policy formulation. For policymakers in high-income countries, there may be a need to reassess the approach towards digital trade barriers, ensuring that they do not stifle the potential benefits of digital integration in value chains. Conversely, in low-income countries, a cautious approach towards rapidly lifting such barriers might be advisable, to allow for the organic development of local industries and their eventual integration into the global digital economy. This comparative analysis suggests a paradigm shift in the understanding of RCDF’s role in global trade, challenging the one-size-fits-all approach and advocating for a more nuanced, context-specific understanding of trade policies.

### Robustness tests

#### Shrinkage procedure

In this study, we’ve applied a 1% tail trimming to all variables, from both ends, to mitigate the impact of outliers. The outcomes of the regression are presented in Table 3. M2 represents a regression conducted for the stability of DVCs. The regression coefficient of RCDF is -0.0302, the findings from the regression analysis indicate that the RCDF coefficient remains significantly negative, thus affirming the robustness of our benchmark regression.

#### Addressing endogeneity

To tackle the endogeneity bias that arises from issues like omitted variables and reverse causality, we’ve employed the instrumental variable technique. This helps to counter potential endogeneity problems within the econometric model. We’ve chosen the count of e-commerce and digital trade rules within all preferential trade agreements (PTAs) signed by each country as our instrumental variable. Drawing from existing studies, this instrumental variable was chosen because countries (or regions) operating under identical trade rules tend to have more aligned digital trade policies. Furthermore, the more agreements containing digital trade rules a country signs, the higher its level of digital trade openness and the lower its RCDF level, satisfying the relevance assumption. However, the presence of digital trade clauses in the signed PTAs does not affect the stability of DVCs, which satisfies the exogeneity assumption. We’ve constructed the instrumental variable in a specific manner to enhance its effectiveness.


$$IV_{\theta jt}=1/(DTR_{\theta jt}+1)$$ (5)

In Eq (5), *IV*_*θjt*_ denotes the RCDF instrumental variable for country *θ*, while *DTR*_*θjt*_ represents the count of PTAs that country *θ* has signed, which include digital trade rules. The data is derived from the statistics on specific agreement terms of PTAs as reported in the TAPED database [68] and the official RTA database of the WTO.

Table 4 displays the outcomes of the endogeneity treatment post tail trimming. The Kleibergen-Paap rk LM statistics stand at 233.799 and 241.421 respectively, and are significant at the 1% level, thereby rejecting the null hypothesis of insufficient identification of the instrumental variable. The Cragg-Donald Wald F statistic surpasses the critical value at the 10% level, confirming the absence of a weak instrumental variable. Hence, we can conclude that the instrumental variable is effective. The regression results reveal that the RCDF coefficient is significantly negative, suggesting that even after employing the instrumental variable to address the endogeneity issue, RCDF continues to significantly suppress the stability of DVCs. This is in line with the findings from the benchmark regression.

**Table 4:** Endogenous treatment: instrumental variables approach.

Variables	*lnDVCs*	*lnDVCs*
		High-income group
	M5	M6
*lnRCDF*	-0.472*** (-2.85)	-0.503*** (-3.42)
*lnFDIR*	-0.0164*** (-4.00)	-0.0188*** (-3.94)
*lnED*	0.736*** (5.11)	1.255*** (4.59)
*lnDTD*	-0.847** (-2.06)	-1.639*** (-3.19)
*lnTO*	2.864*** (5.02)	4.315*** (5.83)
*lnFO*	1.044 (1.46)	1.468* (1.81)
_cons	-0.847** (-2.06)	-12.97** (-2.14)

Kleibergen-Paaprk LM	233.799 [0.0000]	241.421 [0.0000]
Cragg-Donald Wald F	135.500	170.817
Kleibergen-Paaprk Wald F	231.527 [16.38]	231.709 [16.38]
Year	Yes	Yes
Country	Yes	Yes
Industry	Yes	Yes
N	11137	9486
R-sq	0.444	0.449

Notes: (1)*, **, *** represent at 1%, 5%, and 10% significance levels, respectively; (2)The figures in () indicate the t-values;the figures in [] are the Stock-Yogo critical value at the 10% significance level.

As the results in Table 4 show, the coefficient of the RCDF for high-income countries in M6 is -0.503, which is significantly negative and consistent with the results of the heterogeneity test. This result proves that after dealing with endogeneity, the test results for high-income countries remain robust.

### Mechanism testing

This paper constructs a mediation model with innovation capacity as the mechanism variable to examine the pathways through which digital services trade affects domestic value chain quality. Following the steps proposed by [69] to test mediation effects, the models are set as follows:

$$DVCs_{\theta it}=\alpha_{0}+\alpha_{1}RCDF_{\theta it}+\alpha_{2}\mathrm{Controls}+v_{\theta }+v_{i}+v_{t}+\varepsilon_{\theta it}$$ (6)

$$Innov_{\theta it}=\beta_{0}+\beta_{1}RCDF_{\theta it}+\beta_{2}\mathrm{Controls}+v_{\theta }+v_{i}+v_{t}+\varepsilon_{\theta it}$$ (7)

$$DVCs_{\theta it}=\delta_{0}+\delta_{1}RCDF_{\theta it}+\delta_{2}Innov_{\theta it}+\delta_{3}\mathrm{Controls}+v_{\theta }+v_{i}+v_{t}+\varepsilon_{\theta it}$$ (8)

where, *Innov*_*θit*_ is the mediating variable representing country *i*’s innovation level in year t. The proxy variable is from the Global Innovation Index (GII). Since the index contains 7 sub-indices, the 6th and 7th sub-indices on knowledge and technology outputs (Tech) and creative outputs (Crea) directly measure a country’s technological innovation. Thus, this paper uses the ratio of knowledge, technology and creative outputs to total innovation outputs (Innout) as a proxy indicator of technological innovation level, calculated as:

Innov=(Tech+Crea)/Innout (9)

The regression analysis for Eqs (6), (7), and (8) is methodically explored, with the mediation effect model’s results detailed in Table 5. Specifically, in M7, the regression coefficient is -0.00375, which is statistically significant at the 1% level. This significant negative coefficient indicates that digital service trade barriers have a considerable inhibitory effect on innovation capacity. Moving to M8, the mediating variable’s coefficient stands at 0.219, which is also significant but in a positive direction. This suggests that RCDF negatively impact the stability of DVCs by curbing innovation, echoing the effects observed with localization trade barriers in traditional trade contexts [70].

**Table 5:** Mechanism test: mediating effect.

Variable	ln *Innov*	*lnDVCs*	*lnDVCs*		
			High-income	Low-income	
	M7	M8	M9		M10
*lnRCDF*	-0.00375*** (-5.11)	-0.0389*** (-3.01)	-0.0352*** (-2.65)		0.0521 (0.13)
ln *Innov*		0.219** (2.34)	0.337*** (3.03)		-0.130* (1.68)
Controls	Yes	Yes	Yes		Yes
Year	Yes	Yes	Yes		Yes
Country	Yes	Yes	Yes		Yes
Industry	Yes	Yes	Yes		Yes
N	11235	10865	9452		1447
R-sq	0.380	0.934	0.385		0.434

Note:

***, **, and * indicate significant at the 1%, 5% and 10% levels, respectively, with T-values in parentheses.

The coherence in the signs of these coefficients is noteworthy. It implies that technological innovation acts as a partial mediator between RCDF and DVCs, thus lending support to Hypothesis 3. The implementation of RCDF within a country can obstruct the domestic spillover of new technologies, thereby suppressing domestic innovation. This suppression extends to various facets of innovation within the value chain, including product, process, technological, and service innovation. Consequently, this leads to a negative impact on the production efficiency and the value enhancement of intermediate goods. Such an outcome counteracts the potential improvements in DVCs stability that could otherwise be realized through enhanced innovation capacity.

This study also aims to dissect the mechanism of action of RCDF in high-income versus low-income countries. In M9, which focuses on high-income countries, the regression coefficients for β1 and δ2 of -0.0352 and 0.337, respectively, and both are significant at the 1% level. These findings indicate that an increase in RCDF in high-income countries suppresses domestic innovation capacity and therefore affects the stability of DVCs. In contrast, when examining low-income countries, the mechanism of action appears to be insignificant. This suggests that the role of digital factors in facilitating technological innovation varies considerably depending on the country’s stage of technological development, as well as the extent of digital segregation and integration adopted by different countries.

For high-income countries, digital technologies can assist in building national systems of innovation and are a natural element in consolidating technological and innovation advantages within the country. Therefore, RCDF can hinder the healthy and stable development of their value chains. This aligns with the observations made by Baldwin and Tomiura (2020) [1]. Conversely, in low-income countries, the potential enabling role of digital technologies and their transmission are constrained by the limitations in innovation capacity. Thus, the impact of RCDF in these countries does not follow the same pattern as in their high-income counterparts.

## Discussion

This scholarly investigation thoroughly explores the functional mechanism of RCDF on DVC stability and conducts a heterogeneity analysis across various income levels. It is discerned that RCDF generally reduces the stability of DVCs, thereby increasing industrial correlation risk. This finding extends the research scope of Saberi et al. (2019) [71] into digital technology’s role in supply chain management.

The income level is identified as a critical factor in the disparate impacts of digital trade regulatory policies. In affluent regions, RCDF notably suppresses DVC stability, whereas in less affluent areas, its impact is less pronounced. Interestingly, lower-income regions may use RCDF to protect domestic industries and data security, potentially enhancing efficiency within their value chains.

Mechanism analysis shows that RCDF hampers technological innovation. Wealthier nations, with advanced digital technologies, face greater challenges from RCDF, while less affluent countries need to bolster innovation to utilize digital technology effectively.

Despite the insights offered, this study acknowledges room for further enhancement, primarily due to constraints in data availability and associated index measurement methodologies. Firstly, this study has not managed to strike a balance between considerations of data security and the extent of digital trade openness. There is a lack of consensus on the measurement standards for data security, and this study hypothesizes that as the momentum of digital economy development quickens, the repercussions of digital trade openness will also amplify. Secondly, due to data accessibility limitations, RCDF has not been further dissected by industry. The analysis of industry heterogeneity is instrumental for a profound understanding of the operational mechanism of digital trade policies. As data accessibility improves, more comprehensive and expansive research perspectives will gain increasing significance, marking the future research trajectory of this study.

## Conclusion and implication

### Conclusion

Our study provides a comprehensive analysis of the impact of the RCDF on DVCs across various economic contexts. We find that “digital trade openness”, characterized by unrestricted market access and receptivity to foreign digital services, is increasingly crucial for fostering innovation capacity. However, our findings reveal a differential impact of RCDF on low-income versus high-income countries. In low-income countries with less developed technological infrastructures, the adverse effects of RCDF are less pronounced, and in some cases, RCDF may even facilitate the integration of DVCs into GVCs, enhancing stability. Conversely, in high-income countries, we observe a more significant negative impact, necessitating proactive approaches to mitigate these effects.

### Implications

For low-income countries, we recommend the implementation of stringent FDI policies focused on technology transfer and skill development, alongside prioritizing economic growth and trade openness. These strategies are essential for protecting emerging industries and fostering gradual integration into the digital economy.

For high-income countries, these nations should lead in establishing equitable digital trade norms and invest in digital infrastructures that support all sectors. Promoting financial openness and fostering global digital security and trade partnerships are critical steps to counter the negative impacts of RCDF.

In short, tailored digital policies are paramount. Balancing the protection of domestic industries with the integration into global digital trade requires dynamic and adaptable policies. Global collaboration to harmonize standards and practices in digital trade and FDI regulation is crucial for the benefit of global value chains.

### Further research

Based on the findings of the current study, we plan to undertake a comprehensive approach to further understand the impact on other attributes of the value chain. This will include a longitudinal analysis to assess the long-term effects of RCDF, with a focus on the evolving nature of digital trade policies and technological advancements. We aim to explore how different economic sectors within both low-income and high-income countries uniquely respond to these dynamics, seeking to uncover nuanced impacts and provide sector-specific insights. A key part of this analysis will involve evaluating the effectiveness of policy interventions aimed at mitigating the effects of RCDF, using comparative analyses to identify best practices across varied economic contexts. Furthermore, we will investigate the role of emerging digital technologies, such as blockchain and AI, in reshaping DVCs and GVCs, particularly their potential in enhancing efficiency and driving innovation. This integrated research approach is designed to deepen our understanding of the complex interplay between digital trade, policy, and regional value chains.

## Supporting information

S1 Data (DTA)
